# Ranson's Criteria for Acute Pancreatitis in High Altitude: Do they Need to be Modified?

**DOI:** 10.4103/1319-3767.37797

**Published:** 2008-01

**Authors:** Saeed A Abu-Eshy, Mostafa A. Abolfotouh, Eldawi Nawar, Abdul-Rahman H Abu Sabib

**Affiliations:** Department of Surgery, College of Medicine, King Khalid University, Abha, Saudi Arabia; * Family and Community Medicine, College of Medicine, King Khalid University, Abha, Saudi Arabia; ** Department of Surgery, Aseer Central Hospital, Abha, Saudi Arabia

**Keywords:** Acute, high altitude, pancreatitis, Ranson's criteria, Saudi Arabia, severity prediction

## Abstract

**Background/Aim:**

To examine the validity of Ranson's criteria in the prediction of the severity of acute pancreatitis (as judged by the occurrence of complications) in a high-altitude area of Saudi Arabia with a predominant biliary pancreatitis.

**Materials and Methods:**

All consecutive cases of acute pancreatitis (AP) admitted to a tertiary care hospital over a two-and-half-year period were included in this prospective study. Ranson's criteria (RC) were used to determine the severity of the attack of AP, which was then correlated with the occurrence of complications. The validity of Ranson's score and that of each of its individual components was estimated. Using receiver operating characteristic (ROC) curve, new optimum values for these components were calculated and a new modified score was constructed.

**Results:**

Seventy-three attacks of AP in 69 patients formed the material of this study. Ranson's prediction criteria classified 43.8% of the attacks as “severe”, but only 22% of those attacks were associated with complications. Calcium level (<8 mg/dl) was the only criterion that was significantly associated with complications (Kappa = 0.32, *P* = 0.02). Using ROC curve to determine the optimum cut-off levels for prediction identified only four criteria, which were significantly associated with complications as compared with the original Ranson's cut-off levels. Those were: a serum glucose value of ≥160 mg/dl (*P* < 0.05), blood urea nitrogen rise of ≥35 mg/dl (*P* < 0.02) and an arterial Po_2_ value of ≤55 mm Hg (*P* < 0.01), in addition to calcium value of <8 mg/dl (*P* = 0.02) as originally set by Ranson. A new scoring system, ranging from 0 to 4, based on these cut-off levels, together with a calcium level of <8 mg/dl, could correctly classify the severity of AP. A total score of two or more points predicted a severe attack with a sensitivity of 88%, a specificity of 82% and a Kappa coefficient of 0.47 (*P* < 0.001).

**Conclusion:**

This study showed that Ranson's criteria may need to be modified in high altitude with a predominant biliary pancreatitis in order to accurately predict the severity of AP.

An early prediction of severe acute pancreatitis (AP) is essential for institution of appropriate treatment and amelioration of the outcome. Although the scoring systems of Ranson and Imrie could be considered the best available systems for prediction of the severity in AP, especially in alcoholics, there is a need to modify these systems in other clinical settings.[1–5] Since Ranson and colleagues in 1974 identified 11 prognostic factors, considerable research had been undertaken to find the ideal predictor(s) that allow rapid and correct assessment of the severity of AP to suit different clinical and regional settings.[6,7]

The region of Aseer is a mountainous terrain, with a population of 1,200,000 and covers more than 80,000 km^2^ in Southwestern Saudi Arabia. Sharing its Southern border with Yemen, the area extends from the high Aseer Mountains, almost 3200 m (9600 feet) above sea level, down to the Red Sea. Abha, the capital city of the region (population = 1,22,000), is on the Aseer mountains, which is 3133 m above sea level; it has the lowest mean annual temperature compared with any of the southern urban areas, a low atmospheric pressure of oxygen and a high annual rainfall with rain falling mainly in winter and spring. Biliary pancreatitis is the predominant type of pancreatitis.[8] Therefore, this study was conducted to analyse the usefulness of Ranson's criteria (RC) in predicting the severity of AP in this setting of high altitude with scarce alcohol consumption and predominant biliary AP.

## MATERIALS AND METHODS

During a two-and-half-year period, 73 consecutive cases of acute pancreatitis were admitted and treated at Aseer Central Hospital. The diagnosis of AP was based on the presence of typical abdominal symptoms and a serum amylase level above 1000 units per litre. The clinical and biochemical data during the course of the attack were studied and recorded, together with the management and the outcome. Abdominal Ultrasound (US) and/or computed tomography (CT) scanning to detect pancreatic collections, necrosis and cysts were done as necessary. Complications, whether local, such as pancreatic necrosis, abscess or pseudocyst or systemic such as organ(s) failure were detected and evaluated as agreed at Atlanta.[9,10] Severity of AP was categorized based on the clinical and laboratory data using RC [Table 1]. Cases with less than three positive criteria were classified as “mild” and those with three or more positive criteria were classified as “severe”.

**Table 1:** Ranson's criteria: The 11 early objective signs used to classify the severity of acute pancreatitis

Ranson's criteria
At admission or diagnosis
Age over 55 years
White blood count over 16,000/cu mm
Blood glucose over 200 mg/dl
Serum Lactic dehydrogenase (LDH) over 350 U/l
Serum glutamic oxaloacetic transaminase (AST) over 250 U/l
During initial 48 h
Hematocrit fall greater than 10% points
Blood Urea nitrogen rise more than 5 mg/dl
Arterial Po_2_ below 60 mm Hg
Serum calcium below 8 mg/dl
Base deficit >4 meq/l
Estimated fluid sequestration more than 6000 ml

**Data analysis:** The association between complications and Ranson's severity index (mild vs. severe) was tested using the Chi-Squared test or Fisher's exact test (two-tailed) at the 5% level of significance using the SPSS for windows statistical software version 10. The total Ranson's score of each case and the values of different RC were cross-tabulated according to the Ranson's severity index (mild and severe) and these were studied in correlation with the outcome of AP (complicated vs. non-complicated). From these tabulations, the sensitivity, specificity and positive predictive values were computed for each Ranson's criterion. In addition, the level of agreement between the severity according to Ranson and the outcome was determined by the calculation of kappa coefficient.

The receiver operating characteristic (ROC) curve was used for each component of Ranson's criteria to determine the threshold value at which complications of AP could occur.

## RESULTS

During the study period, 73 attacks of AP in 69 patients (four had repeated admissions) fulfilled the diagnostic criteria for entry in this prospective study. In 50 (68.5%) of the attacks, the underlying cause was biliary disease. Alcohol abuse was present in only one patient (1.4%), while 18 (24.7%) patients had unknown underlying cause (idiopathic).

According to RC, 32 (43.8%) of the 73 attacks of AP were classified as severe, although only seven (21.9%) developed complications. Those were pancreatic pseudocyst in two patients (of which one became infected), pancreatic abscess in one patient, chest complications (bronchopneumonia) in two patients and wound infection in two patients.

Table 2 shows the validity of each of RC in predicting the complications of acute pancreatitis in our patients. Only serum calcium level was significantly associated with complications as estimated by kappa coefficient (κ = 0.32, *P* = 0.02), with a high specificity of 83% and a modest sensitivity of 57%. All other criteria failed to attain a significant association. The ROC curve could identify four criteria that were significantly associated with complications. Table 3 shows the threshold values for these four criteria and compares their validity with the original Ranson's cut-off levels. Those were: glucose value of ≥160 mg/dl (*P* < 0.05), BUN rise of ≥35 mg/dl (*P* < 0.02) and arterial Po_2_ value of ≤55 mm Hg (*P* = 0.007), in addition to calcium value of <8 mg/dl (*P* = 0.02) as originally set by Ranson. A scoring system based on these four significant cut-off levels could allocate patients into the range of 0–4 points. A total score of two or more was associated with complications with sensitivity of 88% and a specificity of 82% and a highly significant association of 47% (kappa coefficient, *P* < 0.001).

**Table 2:** Validity of some of Ranson's criteria for prediction of the outcome in acute pancreatitis

Criteria	Sensitivity	Specificity	PPV	NPV	Kappa	Significance
Age	0.50	0.65	0.15	0.91	0.07	0.42
WBC	0.38	0.88	0.27	0.92	0.22	0.06
Glucose	0.13	0.95	0.25	0.90	0.10	0.36
LDH	1.0	0.15	0.17	1.0	0.05	0.24
AST	0.25	0.69	0.10	0.88	-0.036	0.722
HCT%	0.25	0.68	0.10	0.86	-0.043	0.683
BUN	0.29	0.92	0.33	0.91	0.223	0.086
Calcium	0.57	0.83	0.36	0.92	0.32	0.02*
PO_2_	0.63	0.72	0.33	0.90	0.259	0.061

PPV - Positive prediction value, NPV - Negative predictive value.

* Statistically significant at 0.05 level.

**Table 3:** Validity of the modified criteria for predicting the outcome in AP and their corresponding Ranson's criteria

Criteria	Cut-off points	Sensitivity	Specificity	PPV	NPV	Kappa	Significance
Glucose (mg/dl)							
Ranson	>200	0.13	0.95	0.25	0.90	0.10	0.36
Modified	≥160	0.63	0.72	0.22	0.94	0.19	<0.05*
Urea (mg/dl)							
Ranson	>5	0.29	0.92	0.33	0.91	0.223	0.086
Modified	≥35	0.57	0.83	0.31	0.94	0.29	0.015*
Calcium (mg/dl)							
Ranson	<8	0.57	0.83	0.36	0.92	0.32	0.02*
Modified	<8	0.57	0.83	0.36	0.92	0.32	0.02*
PO_2_ (mm Hg)							
Ranson	<60	0.63	0.72	0.33	0.90	0.259	0.061
Modified	≥55	0.63	0.83	0.45	0.91	0.40	0.007*

PPV - Positive predictive value, NPV - Negative predictive value.

* Statistically significant at 0.05 level.

Table 4 compares the original Ranson's score with the modified new score (based on the new four criteria) in terms of validity and the association with complications. The original score had a sensitivity of 88%, a specificity of 62% and a positive predictive value of 22% (κ = 0.21, *P* = 0.008), whereas the new modified score had a similar sensitivity of 88%, but was associated with a higher specificity of 82% and almost doubling of the PPV to 41% and the strength of association with complication was higher (κ = 0.47, *P* < 0.001).

**Table 4:** Sensitivity, specificity, PPV and NPV of both Ranson's score and the new modified score in predicting the outcome of acute pancreatitis

	Complicated	Non-complicated	Sensitivity	Specificity	PPV	NPV	Kappa	*P*-value
Ranson								
>3	7	25	0.88	0.62	0.22	0.98	0.21	0.008
<3	1	40						
Modified score								
>2	7	10	0.88	0.82	0.41	0.98	0.47	<0.001
<2	1	45						

PPV - Positive predictive value, NPV - Negative predictive value.

## DISCUSSION

The ideal predictor of the severity of AP is described as being simple, highly sensitive, highly specific, safe, reproducible, cheap and can be rapidly performed, but unfortunately this ideal predictor does not exist.[7] The multifactorial scoring systems of Ranson and Imrie *et al.*, are accurate in classifying AP in alcoholics rather than gallstone-related pancreatitis.[2,11,12] However, these systems require modification to suit gallstone pancreatitis which is the predominant type in Saudi Arabia.[2–4,8] Imrie *et al.*[13] discarded three of the 11 criteria of Ranson (fluid sequestration >6 l, base deficit >4 mmol/l and haematocrit decrease of >10% within 48 h of admission) and introduced serum albumin <32 g/l to provide a new system based on nine criteria. In their reports, all patients who died were correctly classified as severe by the new scoring system. Balmey *et al.*[3] confirmed the predictive value of only eight of the nine original factors adopted by Imrie *et al.* and showed that the overall predictive value improved from 72% to 79%. However, Osborne *et al.*[2] found that in the subgroup of patients with gallstone-associated pancreatitis, the age factor (>55 years) was not of individual prognostic significance. Also, Leese and Shaw[14] confirmed an improved prognostic performance for modifications of the original Glasgow system. In the present study, RC classified 43.8% of the attacks as severe, but only 22% of these attacks were associated with complications. Al-Qasabi *et al.*[15] from Riyadh, Saudi Arabia, found that despite 66% of cases being classified as severe according to RC, only 36% of this group of patients developed complications. Brisinda *et al.*[5] from Italy found that fever at admission, plasma glucose, blood urea nitrogen, serum creatinine, serum calcium, LDH, serum albumin, red cell count, WBC, haematocrit and lymphocyte count were statistically significant predictors. In view of all these differences, the predictive value of each individual factor must be verified and tested in each clinical and regional setting.

This study revealed that Ranson's score was significantly associated with complications as evidenced by a significant Kappa coefficient. Also, it showed a high sensitivity of 88%, a modest specificity of 62% and a low positive predictive value of 22%. However, when testing each individual component of Ranson's criteria for validity and association with complications, it was surprising to find that only one of these criteria was a significant predictor of complications, whereas all other criteria were not valid for predicting complications. This sub-optimal accuracy could result in misclassification of subjects. Such misclassification may result in either false reassurance or false warning. However, misclassification in the case of Ranson's score was in favour of false warning rather than reassurance, with 25/73 (34.2%) of individuals being misclassified as severe.

To overcome this false warning, the ROC curve was used to obtain the optimum threshold for each criterion. Four significant levels of only four criteria were identified. Those were: blood glucose of ≥160 mg/dl (instead of >200 mg/dl by Ranson), blood urea nitrogen (BUN) rise of ≥35 mg/dl (instead of >5 mg/dl by Ranson), Po_2_ value within 48 h of admission of ≤55 mm Hg (instead of <60 mm Hg by Ranson) and calcium of <8 mg/dl as determined by Ranson. These findings could be justified by the possible change of some biochemical parameters as a result of the high altitude with its associated hypoxia.[16,17] For example, the increase in haemoglobin concentration at high altitude is one of the best known adaptations to altitude hypoxia. Other reported changes are: changes in body fluid compartments, with inappropriate low secretion of anti-diuretic hormone (ADH), reduced plasma volume, elevated basal metabolic rate by about 2%, weight loss and negative energy balance and lowered fasting blood glucose level (possibly due to its lower absorption) than at sea level.[16] All these possible changes might justify the inappropriateness of Ranson's criteria in the prediction of severity of AP in our situation.

Thus, when a scoring system was applied utilizing those four criteria (with one point assigned for each criterion), a highly significant association with complications was detected at a total score of two or more. At this threshold, specificity improved from 62 to 82%. Thus, the problem of false warning by Ranson score could be reduced from 38% to only 15.9% (10/63). Meanwhile, the relatively low positive predictive value clearly indicates that there is room for improvement in the accurate prediction of severity for pancreatitis in individual patients.

In conclusion, the use of the current Ranson's criteria for the prediction of the outcome of AP may not be valid in our setting of high altitude. A suggested modified criteria and score based on the new cut-off levels for only four criteria would be more reliable and simple, with high validity and accuracy. However, in order to have any confidence in this proposed scoring system, it should be tested on a fresh group of patients at sea level, so that its predictive value can be evaluated, i.e. there is a need to test whether these criteria stand up to scrutiny in any area of Saudi Arabia.
